# Dynamic Quadrupole Selection to Associate Precursor Masses with MS/MS Products in Data-Independent Acquisition

**DOI:** 10.1021/jasms.5c00110

**Published:** 2025-08-08

**Authors:** Keaton L. Mertz, Lia R. Serrano, Pavel Sinitcyn, Joshua J. Coon

**Affiliations:** † Department of Chemistry, 5228 University of Wisconsin-Madison, Madison, Wisconsin 53706, United States; ‡ Morgridge Institute for Research, Madison, Wisconsin 53515, United States; § Department of Biomolecular Chemistry, 5228 University of Wisconsin-Madison, Madison, Wisconsin 53706, United States

## Abstract

Data-independent acquisition (DIA) mass spectrometry facilitates high-throughput, reproducible bottom-up proteomic analyses. Typically, DIA methods coselect multiple precursor ions within a wide selection window. These precursors are simultaneously fragmented, superimposing the product ion signals into a complex chimeric spectrum. A method for varying the quadrupole selection width over the ion accumulation period is described. This method couples the intensity of a product ion to the mass of its precursor ion. By overlapping consecutive selection windows, scan-to-scan product ion intensity profiles can be used to infer precursor mass. We assess the method’s sensitivity to quadrupole width, accumulation time, and mass-to-charge range using internal fluoranthene calibrant and FlexMix calibration solution with Q-Orbitrap configured mass analyzers. Additionally, we explore usability of the described technique on a tryptic-digest monoclonal antibody sample, including both direct infusion and liquid chromatography of the sample. With direct infusion, product ions from two precursors separated by 1 thomson (Th) are resolved with this method using 10 Th windows with 5 Th overlap. The product ions are associated within 0.3 Th of their respective precursor ion’s *m*/*z*. Therefore, product ion spectra have a precursor ion *m*/*z* resolving power of ∼33.

## Graphical abstract

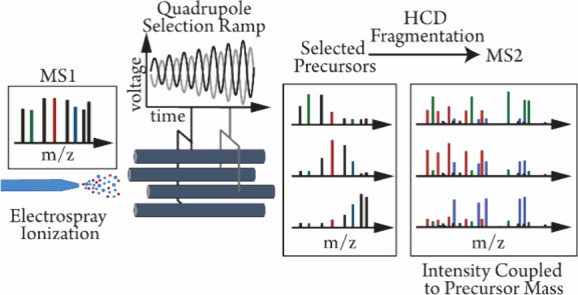

## Introduction

Data-independent acquisition (DIA) mass spectrometry (MS) has gained prominence in high-throughput shotgun proteomics. DIA methods typically employ wide selection windows that coselect and subsequently fragment multiple precursor ions simultaneously.^1–3^ This approach can enhance sampling efficiency, boost the number of detected proteins, and improve run-to-run reproducibility by capturing a broader array of peptides in each cycle.^4,5^ However, the method also can increase the number of chimeric spectra, tandem MS/MS scans with product ions from multiple precursor *m*/*z* peaks intermingled.^6^ The resulting spectral complexity necessitates the use of multidimensional data analysis strategies to confidently identify and quantify peptides.

Product ion signals are often paired with additional dimensions such as retention time,^7,8^ ion mobility,^9^ or MS1 measurements,^10^ thereby aiding in the demultiplexing of these mixed spectra. By correlating the retention time of product ions with that of the intact precursor, or by exploiting precursor ion mobility, these approaches can more effectively separate product ion signals and assign products to the correct precursor ions. While narrowing the DIA selection windows can reduce spectral complexity and improve selectivity, it also increases the number of scans required to cover the entire precursor *m*/*z* range. This trade-off restricts the use of narrow windows to instruments that combine high sensitivity with rapid acquisition speeds, such as time-of-flight (ToF) systems.^11^

The Scanning SWATH method utilizes the rapid acquisition speeds of a SCIEX Q-TOF instrument with a continuously scanning quadrupole.^12–14^ In this method, the precursor selection window maintains a constant width while the central *m*/*z* of the window is ramped at a rate of ∼1 thomson (Th) per millisecond. Product ions are observed in the spectra when their corresponding precursors enter the scanning quadrupole selection window and disappear once the window has passed the precursor. This creates a so-called “Q1” dimension, linking product ion appearance and disappearance to precursor *m*/*z*, thereby providing an additional axis that improves precursor identification.

Building on these advances, we introduce here a dynamic quadrupole selection method that varies the selection window during the ion accumulation period of a scan. This method may find particular application toward instruments with slower acquisition rates, as these instruments rely on wide window DIA to reduce cycle time. By modulating the selection window, precursors near the edges are accumulated for only a portion of the total accumulation time, while the precursors at the center are accumulated for the full duration—forming a triangular-shaped selection window. Upon fragmentation, the resulting product ions inherit an intensity bias from their accumulated precursors. Our method leverages the product ion intensity profile observed across overlapping windows to directly associate product ions with their corresponding precursors. This strategy achieves precursor mass accuracy within 0.3 Th. Importantly, since the precursor mass accuracy is derived solely from the quadrupole’s dynamic behavior, the dynamic quadrupole selection approach minimizes reliance on MS1 scans and is independent from LC elution and ion mobility.

In our study, we provide a proof-of-concept for dynamic quadrupole selection DIA using 15k Orbitrap resolution and a 30 ms maximum accumulation time, settings that enable a sampling rate of approximately 25 Hz in a DIA analysis. We validated our dynamic quadrupole selection method using direct infusion of a monoclonal antibody digest, demonstrating its potential applicability to complex proteomic samples. Furthermore, we demonstrate that the method is compatible with liquid chromatography-based separations.

## Experimental Section

### Materials

Trastuzumab monoclonal antibody was purchased from MilliporeSigma (Cat. MSQC22). The antibody was digested with a rapid-digestion trypsin/lys-C kit from Promega (Cat-VA1061). The digest occurred by the recommended protocol from Promega with a 1:15 enzyme/substrate ratio and a 60 min digestion time. The digested antibody was diluted to 5 μM in 50% methanol and 0.2% formic acid in water for chip-based electrospray mass spectrometry from an Advion Triversa Nanomate. Pierce FlexMix Calibration Solution was purchased from Thermo Fisher Scientific (cat. A39239).

### Mass Spectrometry

All spectra were acquired on an Orbitrap Eclipse Tribrid Mass Spectrometer from Thermo Fisher Scientific. Adjustments to the instrument’s control code allowed for the implementation of the dynamic quadrupole selection. The modification to the control code allowed for the quadrupole rod voltage (the RF and DC component) to be linearly scanned between the starting selection width to the quadrupole selection apex. This linear slew was synchronized to the split-gate timing. Automatic gain control was performed with a static quadrupole window in the linear ion trap to inform each accumulation time for the dynamic quadrupole selection scans (AGC prescan). The standard suite of quadrupole calibrations were performed using FlexMix calibration solution before each experiment unless otherwise stated. All spectra were acquired with a maximum accumulation time of 30 ms with a 100% automatic gain control target unless otherwise stated. The spectra were acquired with the Orbitrap mass analyzer at 15k resolution. These settings were chosen for a fast scan rate of the instrument that would be comparable with other DIA methods.

The fluoranthene cations were generated by the internal EASY-IC Townsend discharge source. These ions were chosen because they produce a very stable scan-to-scan intensity and because the ion production rate can vary with the Townsend discharge current. The Townsend discharge current was set to 1.5 μA for accumulation times less than 5 ms, 0.5 μA for between 5 ms and 10 ms, 0.3 μA for between 10 ms and 20 ms, and 0.2 μA for accumulation times greater than 20 ms. This allowed the variable accumulation time experiments to contain similar ion populations; ∼100% automatic gain control. The fluoranthene data were collected through a method made in the instrument’s Lua control code. The method collected positive mode, centroid, normal mass range, Orbitrap spectra. Five spectra were collected and averaged per experimental condition. The dynamic quadrupole selection starting window widths ranged from 2 to 15 Th wide by 1 Th increments. The accumulation time was varied from 1 to 30 ms by 1 ms increments. By the end of the accumulation time, the final selection window width was 0 Th wide. The scan selection center was stepped 0.1 Th per experiment from the initial to final value.

$${\mathrm{selection center}}_{\mathrm{initial}}=202.07\;\mathrm{Th}-\frac{\mathrm{window width}}{2}-2\;\mathrm{Th}$$

$${\mathrm{selection center}}_{\mathrm{final}}=202.07\;\mathrm{Th}+\frac{\mathrm{window width}}{2}+2\;\mathrm{Th}$$

Pierce FlexMix Calibration Solution was infused at 5 μL/min using a 500 mL glass syringe from Hamilton and a Fusion 101 syringe pump from Chemyx. The calibration solution was introduced to the mass spectrometer via heated electrospray ionization (OptaMax NG) at 3.6 kV relative to ground and an inlet capillary temperature of 90 °C. The FlexMix data were collected in profile mode with 5 microscans.

The Trastuzumab digest was introduced to the mass spectrometer via an Advion Triversa Nanomate. The chip-based electrospray conditions were optimized to provide 120 s of continuous and stable ion current. A sample volume of 2 μL (∼2 ng) was loaded with 1.6–1.8 kV applied voltage and 0.8 PSIg delivery gas pressure. The ESI current was 120 to 140 nA. The mass spectrometer atmospheric inlet was set to 90 °C. The spectra were collected in profile mode with 4 microscans per Orbitrap spectrum.

### Liquid Chromatography–Mass Spectrometry

Reverse-phase liquid chromatography separations of the Trastuzumab digest were performed on a Dionex Ultimate 3000 UPLC from Thermo Fisher Scientific. The column was made in-house using 1.7 μm ACQUITY UPLC BEH C18 sorbent from Waters (cat. 186002350) packed in a 75 μm inner diameter, 360 μm outer diameter bare fused silica capillary.^15^ Solvent A consisted of 0.2% formic acid (FA) in water and solvent B was 70% acetonitrile (ACN) with 0.2% FA in water. One μL of the Trastuzumab digest was injected onto the column. The eluent was ramped from 0% B to 8% B over 1 min, then ramped to 54% B at 73 min, then the column was washed at 100% B for 5 min. The column was held at 55 °C and the eluent flow rate was set to 350 nL/min. The eluate was introduced into the mass spectrometer by ESI at 1950 V.

The MS1 mass spectra were collected with the Orbitrap mass analyzer at 15k resolution without microscans using a standard ion-trap prescan for AGC control of the MS1 accumulation time. The max accumulation time was set to 22 ms for MS1 and MS2 Orbitrap analysis. The MS2 scans were collected with the Orbitrap mass analyzer at 15k resolution with two microscans. Each MS2 scan was preceded by a single ion-trap AGC prescan with a 1 ms accumulation time. The MS2 selection width was 10 Th wide and were only acquired with selection bins centers from 411 to 427 Th by 3 Th steps. However, the data were subset to 6 Th steps for the analysis of the data. This method produced an approximate 1.8 s MS1 to MS1 cycle time. Due to the use of pre-AGC rather than *predictive-*AGC, the MS2 scans were collected without any parallelization of ion accumulation and Orbitrap ion-transient collection. A more advanced implementation of our instrument control code for this method would need to be devised to take advantage of *predictive*-AGC and scan parallelization.

### Data Analysis

The data analysis was performed with python (3.13.1) scripts and the final manuscript figures were formatted in adobe illustrator (AI 2025). The mass spectrometer raw file data were extracted using PyMsFileReader (MIT License, Copyright (c) 2019 François Allain). A 25 ppm tolerance was used for all mass searches. Interactive Peptide Spectral Annotator (IPSA)^16^ was used to verify the functions in the python scripts.

## Results

Here we describe dynamic quadrupole selection, implemented on an Orbitrap Eclipse, a trapping instrument that accumulates ions for each spectrum. In dynamic quadrupole selection, we vary the quadrupole voltages, *U* and *V*, such that the quadrupole selection window symmetrically narrows from 10 Th to 0 Th across the ion accumulation period, maintaining the same *m*/*z* at the center of the selection window. During accumulation, the quadrupole mass filter is scanned such that the relative accumulation time for an ion of mass-to-charge *m*/*z* is given by the transfer function, *T*. In other words, *T* describes the fraction of the total accumulation period during which a precursor of a given mass-to-charge is transmitted through the quadrupole.

T(mz,c,w)={1−2|mz−c|w,if|mz−c|<w20,if|mz−c|≥w2

In this equation, *c* is the mass-to-charge at the center of the quadrupole selection window, and *w* is the starting width of that window. Both *c* and *w* are determined by the quadrupole voltages, *U* and *V*. Within the selection window | mz – *c*| < $\frac{w}{2}$ , the transmission ramps linearly from 0 (0%) at the edges of the window to 1 (100%) at its center. Outside this window, | mz – *c*| ≥ $\frac{w}{2}$ , the transmission is zero.

Because the observed spectral precursor intensity is the product of *T*( mz , *c*, *w*) and the ion’s extrinsic intensity, any measured signal depends jointly on (a) where *m*/*z* falls within the transmission transfer function and (b) the unknown actual ion abundance (depending on solution concentration, ionization efficiency, and various transfer efficiencies). Consequently, to find the precursor mass-to-charge from the measured intensity, it is necessary to solve for both *m*/*z* and the ion’s extrinsic intensity. By collecting additional spectra under different conditions (e.g., shifting *c* to a new center value), additional equations are introduced that can be used to determine the two unknowns. Provided the precursor’s true *m*/*z* remains within the selection window (*c* ± $\frac{w}{2}$ ), it is possible to solve for *m*/*z*. This assumes that the extrinsic conditions remain unchanged for the precursor ion between the two spectra. Any change or noise, particularly from ESI, will introduce uncertainty in the final *m*/*z* calculation.

In the trivial case of a spectrum with no fragmentation, the *m*/*z* is both the mass-to-charge transmitted through the quadrupole and the observed mass-to-charge in the mass spectrum. In this condition, the quadrupole’s transmission transfer function can be characterized and its ability to solve for mass can be assessed under various operating conditions (e.g., different window widths, accumulation times, and center positions). Reporting the difference between the *m*/*z* predicted by the transfer function and the *m*/*z* measured in the spectrum provides a measure of mass-to-charge accuracy for this method.

When fragmentation is applied, the precursor still passes through the quadrupole as described by the above equation and is subsequently dissociated downstream. Consequently, the observed product ion intensities inherently depend on the precursor’s original transmission through the quadrupole. By solving the same system of equations as described previously, this time utilizing the measured product ion intensities, we can again estimate the precursor *m*/*z*. This method couples product ion intensity directly to precursor mass, enabling the confident assignment of product ions to their corresponding precursors.

The application of this method toward a proteomics DIA method is portrayed for two tryptic peptides of similar mass-to-charge. (Figure 1A-B). Four consecutive DIA scans with dynamic quadrupole selection windows are employed. In this example, each DIA window has a width of 8 Th, with 6 Th overlap between adjacent scans creating four measurements of each precursor. The progressively narrowing selection profile during ion accumulation is illustrated by the gray triangular shapes. Modulation of precursor ion accumulation is depicted by varying intensities (red and blue), while the scan selection centers for each scan are indicated by dashed gray vertical lines (Figure 1C).

**Figure 1:**
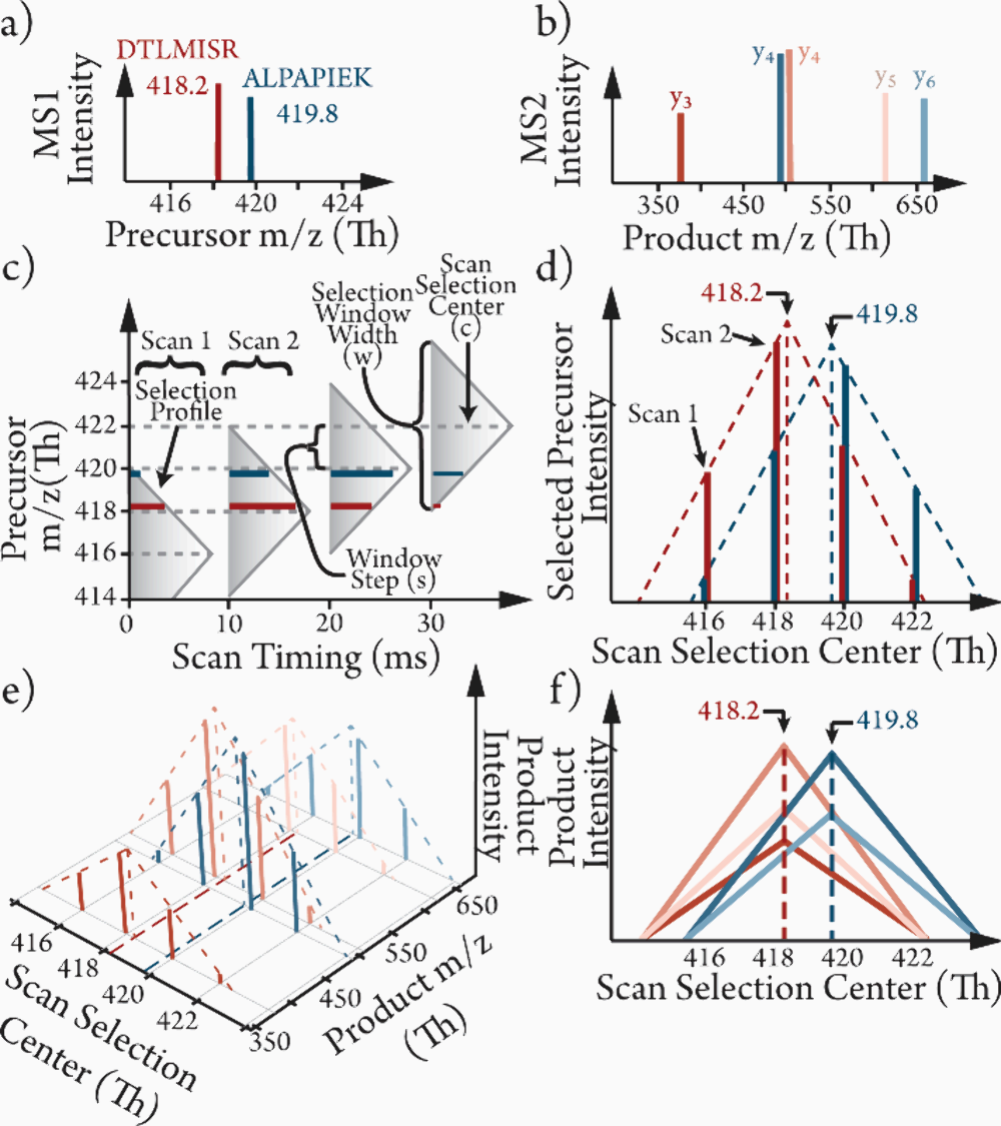
Schematic illustration of the dynamic quadrupole selection method with two precursor peptides. a) MS1 spectrum of two tryptic peptides. b) MS2 spectrum of top five most abundant Prosit predicted^17^ HCD product ions. c) Four consecutive DIA scans with dynamic quadrupole selection. The selection window center for each scan is shown by the dashed gray lines. d) A scan selection center-centric view of the precursor intensities. e) Performing HCD fragmentation on the scans depicted in panel c. f) Dynamic selection product ion intensities are used to determine precursor mass.

Plotting precursor ion intensity as a function of the selection center illustrates how the intensity varies with scan selection center setting. By extrapolating the intensity trend to their intersection, precursor ion’s true mass-to-charge can be identified (the scan selection center that would maximize observed intensity). Notably, the extrapolated x-intercepts span exactly the original 8 Th window width, a crucial detail for scenarios where only two overlapping spectra are available (Figure 1D). Upon precursor dissociation, the observed variation in product ion intensities with the scan selection center further reveals the point at which precursor ion transmission—and thus product ion intensity—is maximized. The intersection point of these extrapolated product ion intensity trends represents the intensity that would be observed if the quadrupole selection window were static rather than dynamic (Figures 1E-F).

To understand the experimental implementation, we simulated an ideal quadrupole to determine the theoretical operational parameters required to achieve the desired quadrupole scanning profile (Figure S1). The simulated quadrupole employed a frequency of 1.1 MHz with rods having a spacing radius (r0) of 4 mm. Simulation results revealed that a 10 Th selection window centered at either 200 Th or 1500 Th would require a similar RF voltage change (∼4.7 *V*_*RF*_) over the ion accumulation period, starting from initial potentials of 277 *V*_*RF*_ and 2109 *V*_*RF*_, respectively. Doubling the selection window width increases the required RF voltage change proportionally to approximately 9.4 *V*_*RF*_. DC potentials exhibited similar behavior as the RF potentials under these conditions. Since the accumulation period defines the time frame for these voltage changes (slew rate), it is critical to characterize this scanning method comprehensively across the entire operational range—including various selection window centers, accumulation durations, and window widths. A nearly constant change in RF and DC voltage in time produced the desired selection profile. Our implementation on the instrument therefore used a voltage profile that changed linearly in time from the starting selection window to the peak of the quadrupole stability.

Automatic gain control (AGC) is critical for the efficient operation of the mass spectrometer. It dynamically adjusts the ion accumulation time to ensure that an optimal number of ions is collected without exceeding the instrument’s charge capacity. To this point, the dynamic selection profile must perform consistently such that scans collected with different accumulation times can be used together to determine the precursor *m*/*z*. Fluoranthene cations produced from the Townsend discharge Easy-IC internal source were used to assess the methods performance over varying accumulation times. Besides stable ion generation (<1% RMS), the internal source intensity can be systematically varied to produce 100% AGC over a wide range of accumulation times (see Experimental section). Fluoranthene spectra was collected for 13 windows widths from 2 to 14 Th and 30 ion accumulation times from 1 to 30 ms. The scan selection center was shifted by 0.1 Th between each scan to produce a highly sampled intensity profiles (Figure 2A and 2B).

**Figure 2:**
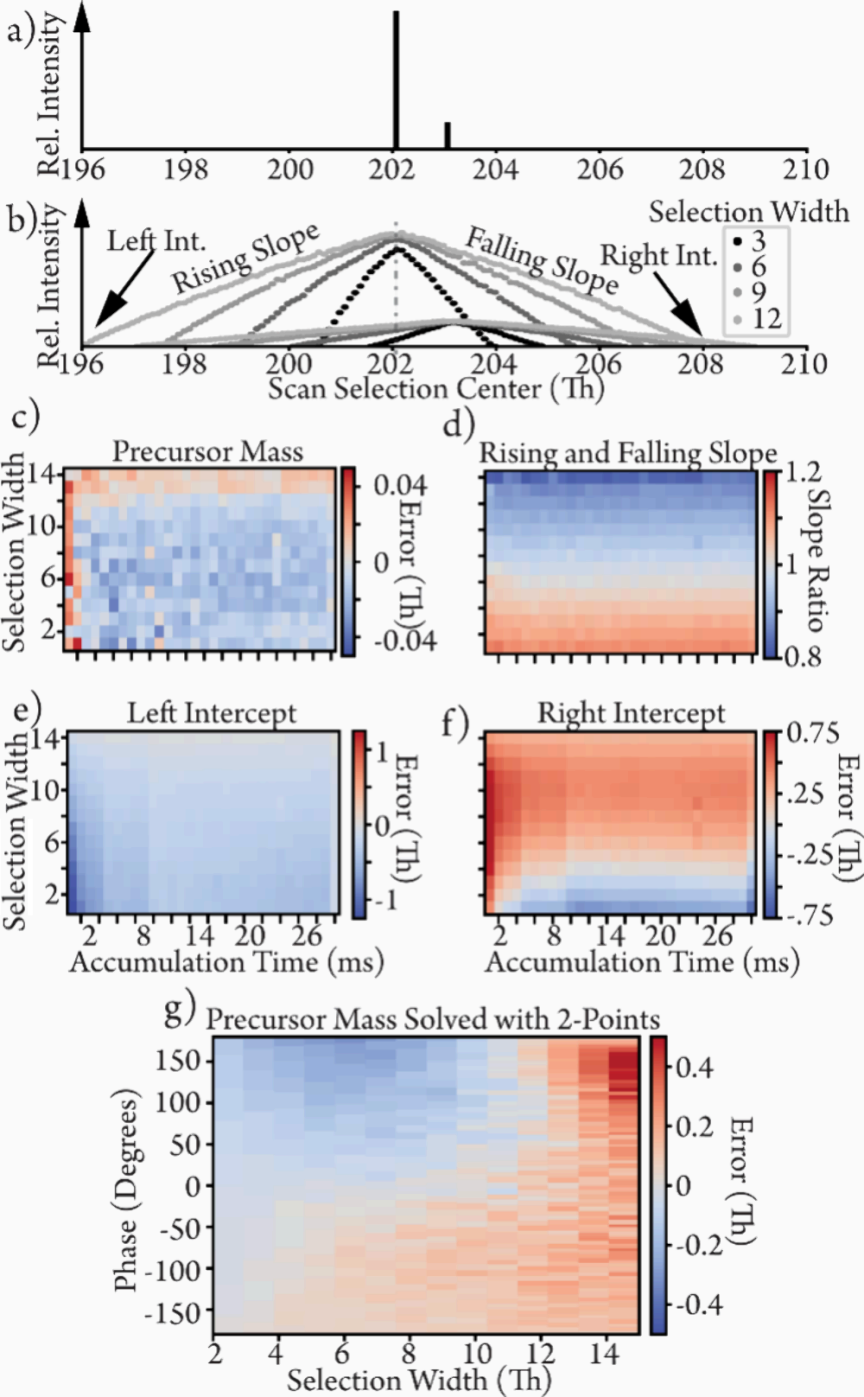
Method validation over selection widths and accumulation times. a) Orbitrap MS1 spectrum of fluoranthene. b) Fluoranthene intensity plotted over scan selection centers. c–f) Deviation of the dynamic quadrupole data from the ideal result. g) The error in calculated precursor mass-to-charge when the data is subsampled to two data points.

Least-squares linear regression of all the 0.1 Th spaced data points was used to validate the empirical transmission transfer function. The deviation of the precursor *m*/*z* calculated from the intersection of the two regressions from the 202.07 Th fluoranthene mass was plotted over accumulation time and window width (Figure 2C). The mass-accuracy of the method is serendipitous as the method can only perform as well as the quadrupole is calibrated, and the quadrupole calibration is not this accurate. However, the potential precession of the method should be demonstrated in the figure. The two x-intercepts are expected to be separated by the selection window width while centered around 202.07 Th. The deviation of the intercepts from this expectation is shown (Figure 2E and 2F). The 10 ms ion accumulation data set was subsampled to extract two data points for each scan selection profile width. The two points were selected so that the two windows would have 50% window overlap. Phase pertains to the relationship between the two data points and the precursor mass. When each data point is equally spaced from 202.07 Th is a phase of 0 deg. If the first scan selection center is at 202.07 then the phase is 180 deg. If the second scan selection center at 202.07, then the phase is −180 deg.

From this experiment we conclude that the method is robust to accumulation time, so the subsequent experiments employed AGC control. Window width had a greater effect on the results, so the trend with respect to window width was plotted. It could be imagined how they could be incorporated into a calibration scheme if greater method precision were desired (Figure S2).

In Figure 2B, a reduction in intensity was observed for acquisitions utilizing smaller selection window widths. It can also be observed that the apex of the profile is slightly rounded rather than coming to a sharp point. We attribute these observations to the reduction in transmission efficiency of the quadrupole at small selection windows. We simulated the effect of a lossy transmission quadrupole (Figure S3). Although the dynamic quadrupole selection profile becomes distorted, the effect on the production association to a precursor *m*/*z* was minimal. The error estimated from the simulation was smaller than that observed in Figure 2G, leading us to the conclusion that there are other significant sources of perturbation to the profile that have yet to be identified.

Pierce FlexMix calibration solution provides signals distributed across the entire mass-to-charge range. Dynamic quadrupole selection was performed from 150 to 1750 Th using selection windows of 10 Th width with 5 Th overlaps, with no dissociation energy (0 NCE HCD) applied. Spectral features observed in multiple consecutive windows were processed to determine their precursor *m*/*z*, which should match the observed spectral *m*/*z*. For the 61 identified features across the mass range, the difference between the two-point calculated precursor *m*/*z* and the observed *m*/*z* was reported. Points were color-coded based on their calculated intensities (Figure 3). Pre-AGC was performed for each window with a maximum accumulation time set at 30 ms, and no correlation between ion intensity and mass error was observed.

**Figure 3:**
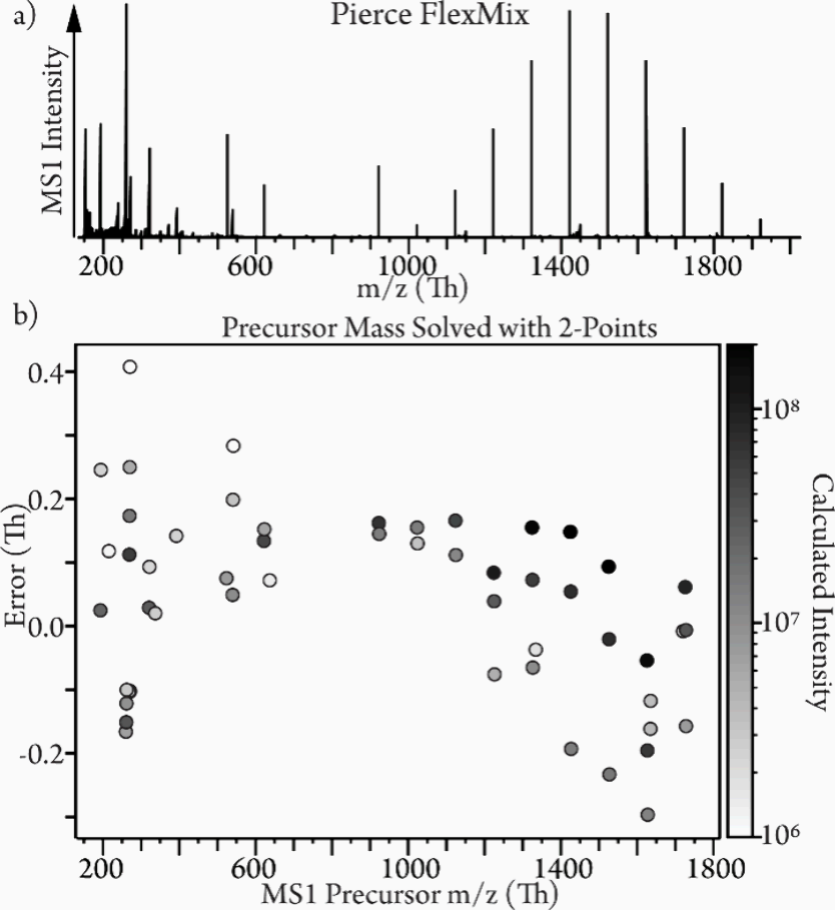
Method validation over mass range. a) Orbitrap MS1 spectrum of Pierce FlexMix. b) Dynamic selection DIA was performed on FlexMix with two data points using 10 Th selection windows with 5 Th overlap. The deviation of the calculated mass from the spectrum mass is shown.

The performance of this method across a broad range of accumulation times, selection window widths, and mass ranges demonstrated that the mass spectrometer electronics reliably executed dynamic quadrupole selections within 0.4 Th without need for modification. This is significant as 0.4 Th is the smallest quadrupole width allowed for DDA analysis. With the method validated we next sought to test its utility for determining precursor *m*/*z* values for product ions. Product ion analysis introduces additional complexity due to products originating from multiple isotopologs of the precursor ions.

To evaluate whether these isotopologs would be problematic, we first modeled the effect on peptides composed exclusively of “averagine” amino acids, excluding sulfur. In Figure 4 we consider a rather large tryptic peptide precursor having 20 amino acids and note (Figure 4A) that indeed the M+1 is the base peak of the isotopic cluster. Such large peptides have a more complex trend in product intensity as a function of the scan selection center. The MS1 scan selection center profile for each isotopolog is shown (Figure 4A). A 3-mer product ion of the 20-mer peptide creates a multifaceted intensity profile with respect to the scan selection center because the profile is nearly the composition of all of the MS1 isotopolog profiles. The extrapolation of this profile identifies the average natural abundance mass as the precursor *m/z* (Figure 4B).

**Figure 4:**
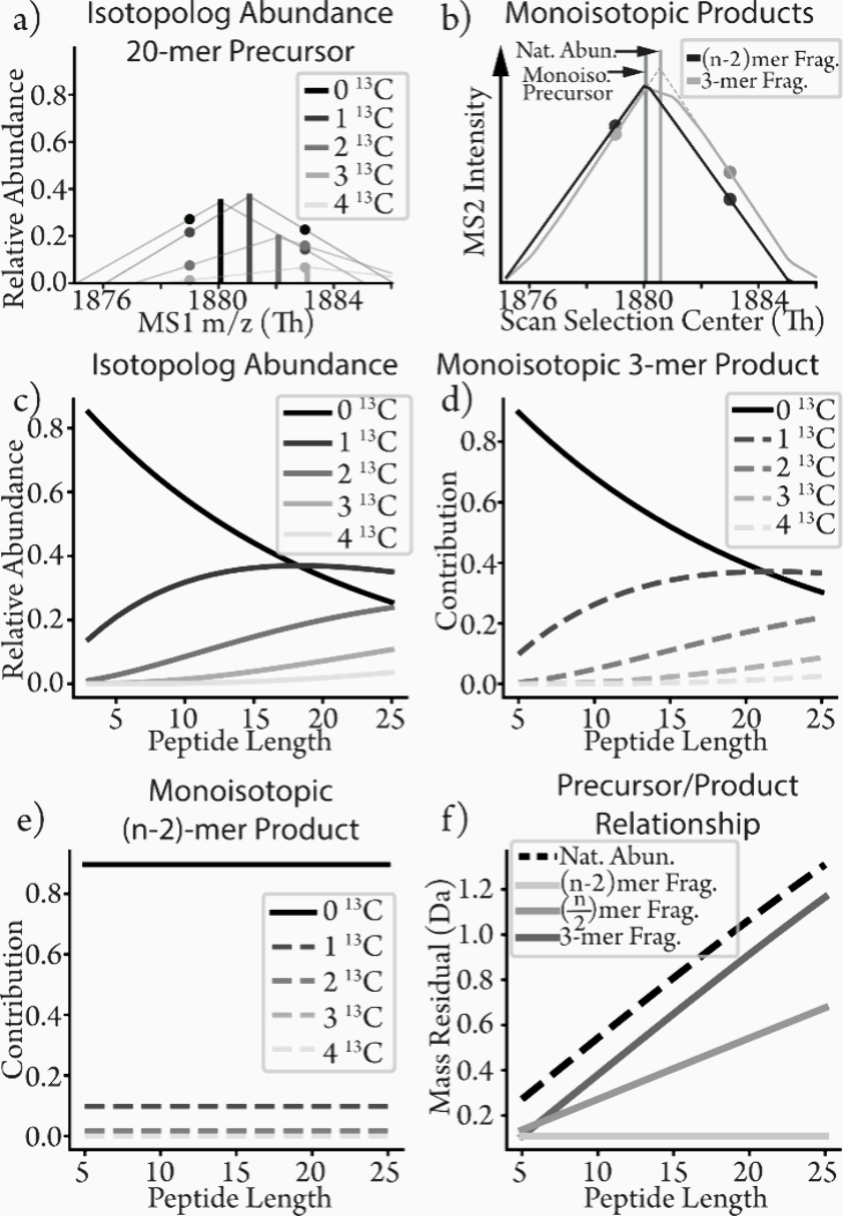
Determining the effect of isotopolog fragmentation on precursor *m*/*z* accuracy. a) Example isotopic distribution and MS1 scan selection center profiles. b) Short and long product ion profiles showing differing profile shapes. c) Isotopolog abundance as a function of peptide length. d, e) Precursor contribution to observed monoisotopic product ion intensity as a function of peptide length. f) Mass residual as a function of peptide length showing the precursor/product relationship.

Next we characterized how the isotopolog distribution of a peptide varies with length, using 1.1% as the natural abundance of the ^13^C isotope (Figure 4C). Smaller peptides predominantly appear as monoisotopic species, allowing their products to directly represent the monoisotopic precursor *m*/*z* value. However, longer peptides contain higher proportions of heavier isotopes, which can significantly contribute to the observed intensity of a monoisotopic product. When a precursor peptide containing one ^13^C isotope is dissociated, the likelihood of the resulting product retaining the heavy isotope depends on the product size: larger products more frequently retain the heavy isotope, while shorter products are more likely to remain monoisotopic.

We quantified the contribution of each precursor isotopolog to the observed intensity of both a monoisotopic 3-mer product and a monoisotopic product that contains the majority of amino acids—an (n-2)-mer product, where n represents the length of the precursor peptide. This analysis considered both isotopolog abundance and the probability of the ^13^C isotope being incorporated into the product (Figures 4D-E). Results indicate that the monoisotopic (n-2)-mer product predominantly originates from the monoisotopic precursor, while the smaller 3-mer product contributions closely follow the isotopic distribution. Consequently, calculated precursor masses based on larger (n-2)-mer products closely align with the monoisotopic precursor mass, whereas those derived from the smaller 3-mer products reflect an average mass or natural abundance mass due to the influence by the isotopolog distribution (Figure 4F). From these data we conclude that the mass discrepancy induced by isotopic is minimal, producing less than a 0.5 Th deviation from the monoisotopic mass in worst-case scenarios, given that most precursors analyzed are shorter and carry charges ≤ 2. Further, for peptides falling outside of this range, or to slightly improve accuracy, one could apply a correction factor.

Having concluded that the presence of isotopes has a minimal impact on precursor ion mass association we next sought to test performance of dynamic selection DIA on a real sample. To do this we prepared a tryptic digest of a monoclonal antibody (Trastuzumab) and introduced the resulting complex mixture of peptides directly into the MS via nano electrospray (nESI, Figure 5). While many DIA methods leverage retention time and chromatography to group product ions with precursors, our dynamic selection method, like Scanning SWATH, can associate product ions without chromatography or ion mobility. Annotated fragmentation spectra from two different selection centers demonstrate clear differences in product ion intensities (Figures 5C-D). These chimeric spectra would look very similar to each other with a static selection window as all of three precursors are inside both windows. Specifically, dynamic selection DIA clearly shows the intensity of peptide sequence GLEWVAR decreasing relative to ALPAKIEK between the two spectra. Three spectra were used to calculate precursor *m*/*z* for product ions, and deviations from actual precursor mass were plotted, yielding a mass accuracy of <0.3 Th for all product ions (Figures 5F-G).

**Figure 5:**
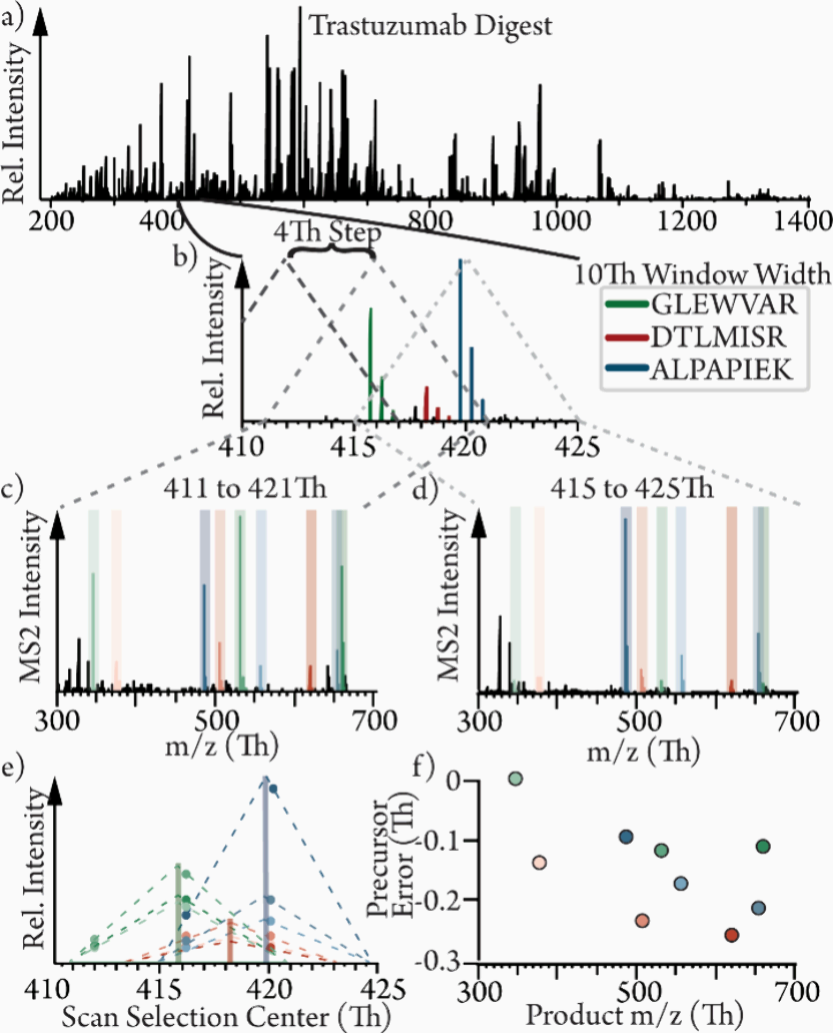
Method validation with direct infusion of a tryptic Trastuzumab digest. a) MS1 of Trastuzumab direct infusion by Nanomate. b) Expanded view of the MS1 spectrum depicting the two 10 Th wide selection windows spaced 4 Th. c, d) HCD fragmentation spectra of the two dynamic selection DIA. e) Extrapolation of two data points to determine precursor *m*/*z*. f) The mass-to-charge difference between the calculated precursor *m*/*z* and the actual *m*/*z* for each product ion.

The Trastuzumab monoclonal antibody digest was analyzed using reversed-phase liquid chromatography (LC). Here the Orbitrap mass analyzer collects spectra at approximately 25 Hz (15k resolution, 30 ms max accumulation time), allowing two consecutive overlapping spectra to be collected within approximately 40 ms. The chromatographic elution profile typically occurs over seconds; thus, this rapid sampling permits a quasi-static approximation, ensuring accurate intensity profiles of dynamically selected precursors. Both chromatographic and dynamic quadrupole selection dimensions can thus be utilized for precursor-product association. Figure 6 illustrates a representative UHPLC reversed-phase chromatogram for two peptides with 30 s elution profiles, demonstrating the preservation of product ion elution profiles by dynamic selection DIA.

**Figure 6:**
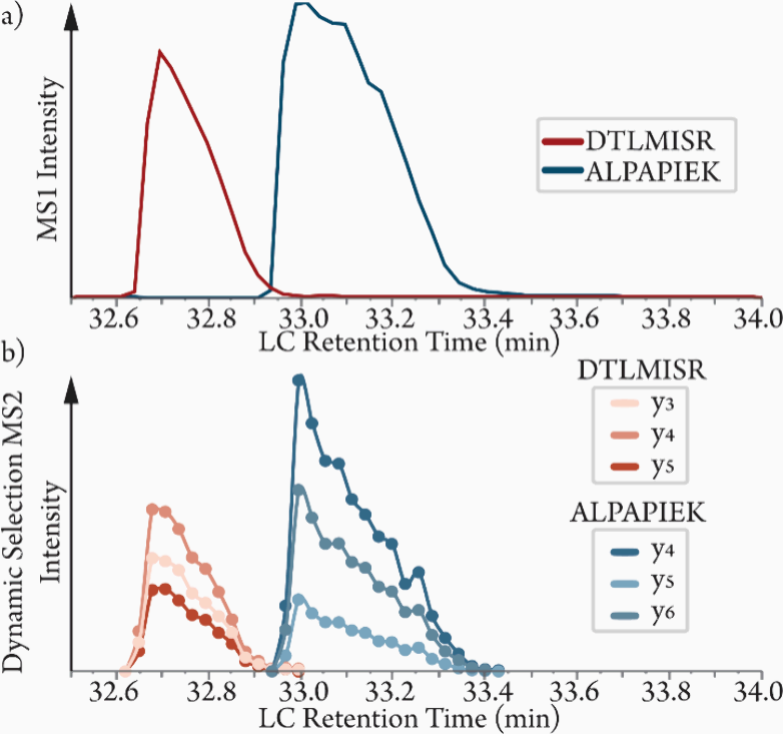
LC Separation. a) MS1 based extracted ion chromatogram for two precursors. b) Dynamic selection DIA product ion chromatogram.

Low-intensity precursors may fall below the detection limit outside their chromatographic peak apex, potentially resulting in insufficient data points for extracted product ion chromatograms. In such scenarios, the rapid successive scans provided by dynamic selection DIA may be able to enable product ions association with their precursor *m*/*z*.

## Conclusion

Here we introduce dynamic selection DIA and demonstrate its ability to accurately associate changes in product ion intensities to their corresponding precursor *m*/*z*. Using this approach we achieved mass accuracy within 0.3 Th for selection windows of 10 Th across a limited set of examples. Therefore, product ion spectra have a precursor resolving power of ∼33. This is a marked improvement over the MSX-DIA method that divides the DIA bins into typically 5 randomly selected 4 *m*/*z* wide selections per scan.^18^ In this way, MSX-DIA allows the deconvolution of complex spectra. The product ions can be associated with precursor *m*/*z* with a resolving power of ∼5.

Results shown here also show compatibility with automatic gain control (AGC), a crucial aspect for practical mass spectrometry applications, allowing dynamic adjustment of ion accumulation times.

Since the method relies on intensity information, one potential challenge of the method is susceptibility to electrospray ionization (ESI) instability, which can negatively impact both label-free quantification and the chromatographic profiles. While microscans were employed to mitigate these stability issues effectively in the current study, such averaging could complicate high-throughput DIA workflows due to increased analysis time requirements. Nonetheless, dynamic selection DIA could function without microscans with careful attention dedicated to optimizing experimental methods and solvent conditions. Further, specialized search software capable of incorporating the dynamic selection dimension into precursor identification scoring algorithms or employing it as a postprocessing enhancement step will be essential for effective analysis of complex biological samples.

Further improvements for future implementations include optimizing AGC parameters. Ideally, through integration directly into instrument software that explicitly accounts for the dynamic selection profile shape. Further, because the selection profile of dynamic selection DIA reduces the average effective ion accumulation time by 50%, as compared to conventional static selection DIA. Thus, on average, the ion accumulation times must be doubled to achieve the same number of ions. This, however, does not necessarily reduce scan rate as ion accumulations time are often shorter than mass analysis times on Orbitrap analyzers, especially for resolving powers of 15,000 and higher. The ion capacity limit is not affected by the method, so a wider selection window could be explored that would accumulate the ACG target fast enough to maintain optimal parallelization of the instrument—although larger selection windows will create more complex product ion spectra. Use of dynamic selection DIA may allow effective searching by deconvolving the complexity in wider selection windows. Implementation of these improvements could achieve greater analytical throughput and sensitivity.

In its current form, dynamic selection DIA is a highly promising method that for accelerating direct-infusion based analysis of complex mixtures. Here we demonstrate this using a mixture of peptides derived from a therapeutic antibody; however, several reports have used direct-infusion for rapid analysis of whole proteomes and even for samples containing peptides, metabolites, and lipids (i.e., multiomics).^19^ And, keeping the considerations noted above, we envision the dynamic selection DIA method could have utility for LC-MS approaches as well.

## Supplementary Material



## Data Availability

The data used in this manuscript as well as the data analysis software are available on GitHub.^20^

## Notes

Targeted selection profile and quadrupole voltages required to achieve the profile, selection profile deviations from expectation for fluoranthene data as a function of selection window width, and the effects of a lossy transmission quadrupole profile (PDF)

## ∇

AI Technology for Life, Department of Information and Computing Sciences, Utrecht University, Utrecht 3584 CC, The Netherlands

## ○

Biomolecular Mass Spectrometry and Proteomics, Department of Pharmaceutical Sciences, Utrecht University, Utrecht 3584 CH, The Netherlands
