# Supplementary material for: Dynamic Quadrupole Selection to Associate Precursor Masses with MS/MS Products in Data-Independent Acquisition
Source: J Am Soc Mass Spectrom. 2025 Aug 8;36(9):1869–76. doi: 10.1021/jasms.5c00110 (PMC12364021; doi:10.1021/jasms.5c00110)
Supplement: Supplementary file 1 [file js5c00110_si_001.pdf]

SUPPORTING INFORMATION

Dynamic Quadrupole Selection to Associate Precursor Masses with MS/MS Products in Data-Independent Acquisition.

Keaton L. Mertz[1], Lia R. Serrano[1], Pavel Sinitcyn[2†*], and Joshua J. Coon[1,2,3*]

[1]Department of Chemistry, University of Wisconsin-Madison, Madison, Wisconsin 53706, United States

[2]Morgridge Institute for Research, Madison, Wisconsin 53515, United States

[3]Department of Biomolecular Chemistry, University of Wisconsin-Madison, Madison, Wisconsin 53706, United States

[†]present address: AI Technology for Life, Department of Information and Computing Sciences, Utrecht University, Utrecht 3584 CC, the Netherlands and Biomolecular Mass Spectrometry and Proteomics, Department of Pharmaceutical Sciences, Utrecht University, Utrecht 3584 CH, the Netherlands
*Correspondence to p.sinitcyn@uu.nl or coon@wisc.edu

Figure S1. Dynamic quadrupole selection voltage requirements S-2
Figure S2. Fluoranthene Calibration Data S-3
Figure S3. Simulating Lossy Quadrupole Transmission S-4

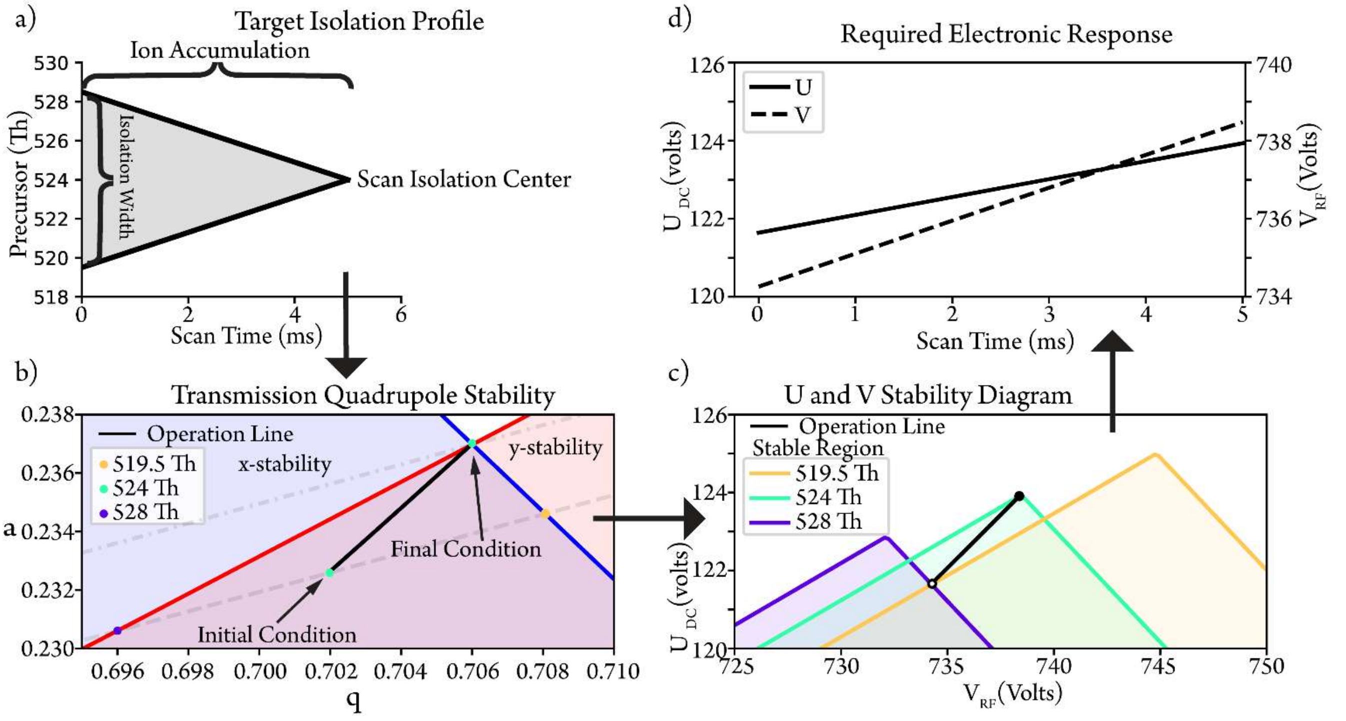

Figure S1. Dynamic quadrupole selection voltage requirements a) The targeted selection profile to encode precursor mass into the accumulated ions of a scan. b) The transmission quadrupole stability diagram in stability terms a and q. To achieve the desired selection profile, the quadrupole settings must travers the black operation line over the ion accumulation period. The 524 Th ion at the scan selection center will remain stable for the full duration of the accumulation while other m/z will only accumulate for a portion of the time. c) The RF and DC potentials required for a 4mm r0 quadrupole operating at 1.1MHz to perform the selection profile. d) The voltage slew in time needed to perform the targeted selection profile.

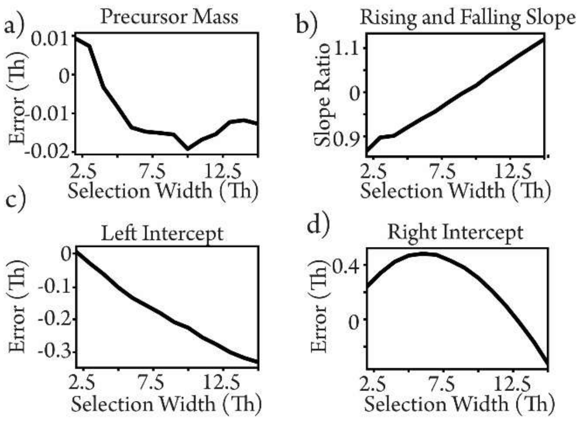

Figure S2. Fluoranthene Calibration Data The heat maps in figure 2 were plotted over dimensions, accumulation time and selection window width. For each heat map, selection window width was found to drive the trend in m/z error. These plots have collapsed the low accumulation time axis via averaging and plots the dominate trend. Data was collected for 13 windows widths from 2 Th to 14 Th and 30 ion accumulation times from 1ms to 30ms. The scan selection center was shifted by 0.1 Th between each scan to produce a highly sampled intensity profile. a-d) Utilizing a least-squared linear regression of all of the 0.1 Th spaced data points, the deviation from the expected intensity profile is shown as measured minus expected for the 202.07 Th fluoranthene.

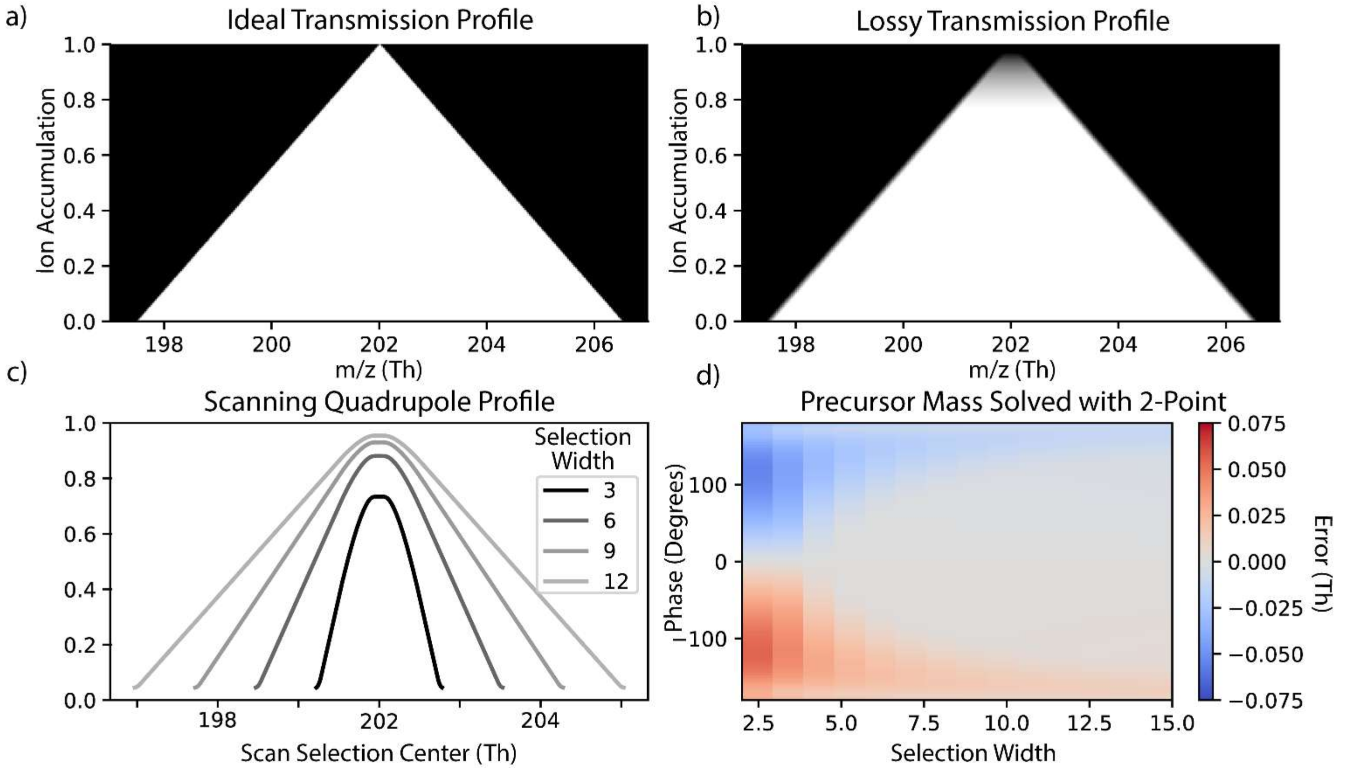

Figure S3. Simulating Lossy Quadrupole Transmission a) Ideal dynamic quadrupole selection profile. b) Simulated transmission profile of a dynamic quadrupole selection scan. The quadrupole is modeled with a trapezoidal transmission profile with a 0.15 Th edge width. Additionally, a decrease in transmission is modeled for narrow quadrupole selection below 2 Th. The equation for the transmission decrease was found empirically to be transmission = 0.5 ln(selection width) + 0.63 . c) The lossy transmission model is integrated to create the dynamic quadrupole selection profiles at four selection widths. d) Selection width 2 Th through 15 Th are modeled in the same style as **figure c.** The data is sub-sampled to extract two data points for each scan selection profile width. The two points are selected so that the two windows have 50% window overlap. Phase pertains to the relationship between the two datapoints and the precursor mass. When each datapoint is equally spaced from 202.07 Th is a phase of 0 degrees. If the first scan selection center is at 202.07 then the phase is 180 degrees. If the second scan selection center at 202.07, then the phase is -180 degrees.
